# An Annular Mechanical Temperature Compensation Structure for Gas-Sealed Capacitive Pressure Sensor

**DOI:** 10.3390/s120608026

**Published:** 2012-06-11

**Authors:** Xiuchun Hao, Yonggang Jiang, Hidekuni Takao, Kazusuke Maenaka, Kohei Higuchi

**Affiliations:** 1 Maenaka Human-Sensing Fusion Project, JST, Shosha 2167, Himeji, Hyogo 671-2280, Japan; E-Mails: yonggangj@gmail.com (Y.J.); takao@eng.kagawa-u.ac.jp (H.T.); maenaka@eng.u-hyogo.ac.jp (K.M.); k-higuchi@eratokm.jp (K.H.); 2 School of Mechanical Engineering and Automation, Beihang University, Xueyuan Road No. 37, Haidian District, Beijing 100191, China; 3 Micro-Nano Structure Device Integrated Research Center, Kagawa University, Takamatsu, Kagawa 761-0396, Japan; 4 Department of Electrical Engineering and Computer Science, University of Hyogo, Himeji, Hyogo 671-2280, Japan

**Keywords:** pressure sensor, mechanical temperature compensation, gas-sealed

## Abstract

A novel gas-sealed capacitive pressure sensor with a temperature compensation structure is reported. The pressure sensor is sealed by Au-Au diffusion bonding under a nitrogen ambient with a pressure of 100 kPa and integrated with a platinum resistor-based temperature sensor for human activity monitoring applications. The capacitance-pressure and capacitance-temperature characteristics of the gas-sealed capacitive pressure sensor without temperature compensation structure are calculated. It is found by simulation that a ring-shaped structure on the diaphragm of the pressure sensor can mechanically suppress the thermal expansion effect of the sealed gas in the cavity. Pressure sensors without/with temperature compensation structures are fabricated and measured. Through measured results, it is verified that the calculation model is accurate. Using the compensation structures with a 900 μm inner radius, the measured temperature coefficient is much reduced as compared to that of the pressure sensor without compensation. The sensitivities of the pressure sensor before and after compensation are almost the same in the pressure range from 80 kPa to 100 kPa.

## 1. Introduction

Compared to widely used piezoresistive pressure sensors [1,2], absolute capacitive pressure sensors are advantageous for their lower temperature drift and low power consumption [3–5]. However, capacitive pressure sensors' vacuum cavities are conventionally sealed by silicon-to-silicon bonding or anodic bonding. These procedures require complex devices, high temperatures and more critical pretreatments. Moreover, such approaches give rise to difficulties in transferring the electrodes from the sealed cavity to the exterior, which has become the main challenge for the design and fabrication of these sensors. Many solutions to this second concern have been introduced [6–8], but their applications are still based on the traditional bonding methods and their corresponding difficulties.

The gas-sealed capacitive pressure sensor is presented here to overcome this problem. The gas-sealed capacitive cavity is bonded by metal diffusion bonding. Using the metal diffusion bonding [9–11], the electrode transfer becomes easier and a high bonding strength can be achieved with a low bonding temperature. The gas-sealed pressure sensors do not need vacuum sealing, especially if the gas pressure of the sealed cavity is under atmospheric pressure, which can make the fabrication easier. In addition, as the objective of this research is to develop an atmospheric pressure sensor for human activity monitoring applications [12], the gas-sealed cavity can be fabricated with a narrower gap between the diaphragm and the substrate, which leads to an increase in the sensitivity around atmospheric pressure. However, the problem in the gas-sealed capacitive pressure sensor is a large thermal drift due to the thermal expansion of the gas inside of the cavity. The conventional temperature compensation method is to use a circuit by measuring the temperature [13]. It is electronically complicated and needs additional integrated circuits. In this paper, a mechanical temperature compensation method using an annular structure is developed [14]. We will describe the design, fabrication and characterization of a temperature compensated gas-sealed capacitive pressure sensor by Au-Au wafer-level bonding for atmospheric pressure measurements.

## 2. Design Process

### 2.1. Gas-Sealed Capacitive Pressure Sensor

**Figure 1.**
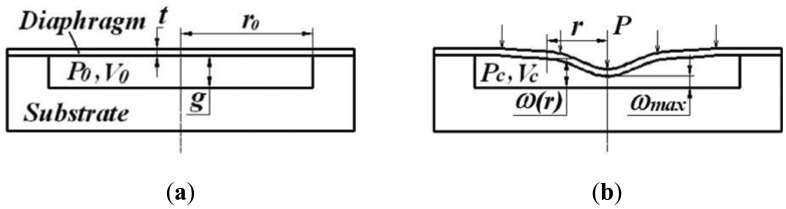
Working principle of the atmospheric pressure sensor. (**a**) in the case that ambient pressure is identical to the initial pressure of the cavity. (**b**) in the case that ambient pressure changes.

The principle of the gas-sealed not-touch mode capacitive pressure sensor is illustrated in Figure 1. The sensor cavity is sealed with nitrogen gas with an initial pressure (*P*_0_). When the ambient pressure (*P*) is identical to the initial pressure of the cavity, there will be no diaphragm deflection and no capacitance change, as shown in Figure 1(a). When the ambient pressure changes, the diaphragm will bend downward or upward due to the difference of the pressure between the ambient air and the sensor cavity, which induces a capacitance change as shown in Figure 1(b). In this case, the inner pressure of the cavity becomes *P*_c_ due to the volume change of the cavity.

A clamped circle thin plate [15] is adopted here as the sensing unit of the capacitive pressure sensor. It is common practice to use the small deflection model if the membrane will deflect less than half of its thickness. Otherwise, a large deflection model would be more accurate. In our design, the thickness of the diaphragm is set to 5 μm, so the large deflection from the plate theory will be more accurate here. The capacitance of the cavity is given by calculating the displacement of the diaphragm with the large deflection model. The centre point deflection of the diaphragm with a pressure difference between the ambient air and the cavity is given by:

(1) $$\omega_{\max}=\frac{(P-P_{c})r_{0}^{4}}{64D}\frac{1}{1+0.488\frac{\omega_{\max}^{2}}{t^{2}}}$$

D is the flexural rigidity, which is defined as:

(2) $$D=\frac{Et^{3}}{12(1-\nu^{2})}$$

where *E* is the Young's modulus of silicon, *ν* is the Poisson's ratio, *t* is the thickness of the diaphragm, and *r_0_* is the radius of the diaphragm. The deflection of the diaphragm at an arbitrary position with a distance (*r*) from the center point is given by:

(3) $$\omega (r)=\omega_{\max}{\left(1-\frac{r^{2}}{r_{0}^{2}}\right)}^{2}$$

The downward deflection of the diaphragm (namely, *P* > *P_c_*) is assumed to be positive in Equations (1) and (3). Using Equations (1) and (3), the capacitance of the cavity can be calculated as:

(4) $$C=\int_{0}^{r_{0}}\frac{{ɛ}_{0}{ɛ}_{r}2\pi r}{g-\omega (r)}dr$$

where *g* is the gap distance between the diaphragm and the substrate, *ε* and *ε_r_* are the permittivity of vacuum and the relative dielectric constant of air, respectively. According to the equation of state of ideal gas, the relation between the initial pressure (*P*_0_) and the pressure (*P*_c_) of the cavity is given by:

(5) $$\frac{P_{0}V_{0}}{T_{0}}=\frac{P_{c}V_{c}}{T_{c}}$$

where *V*_0_, *V*_c_, *T*_0_ and *T*_c_ are the initial volume, current volume, initial temperature and current temperature of the gas sealed in the cavity.

The volume of sealed cavity is calculated by:

(6) $$V_{0}=\int_{0}^{r_{0}}2\pi r(g-\omega_{0}(r))dr$$

(7) $$V_{c}=\int_{0}^{r_{0}}2\pi r(g-\omega (r))dr$$

where *ω_0_*(*r*) is the diaphragm deflection at initial state.

**Figure 2.**
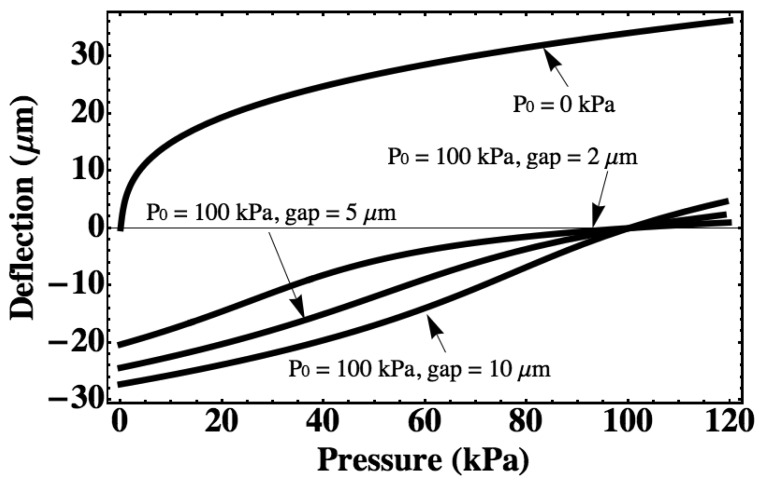
The deflection of diaphragm calculated by large-deflection model with different cavity gap and initial sealed gas pressure. The radius of the diaphragm is 1 mm, and the thickness is 5 μm. The downward deflection is assumed positive, the upward deflection is assumed negative.

**Figure 3.**
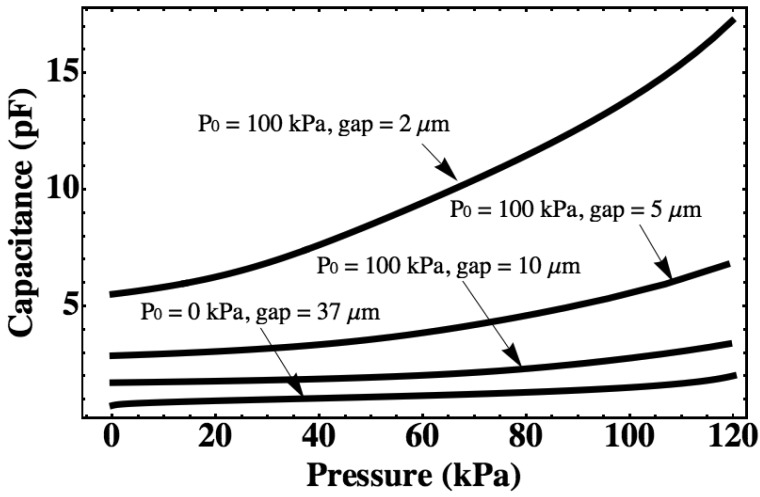
The capacitance of cavity calculated by large-deflection model with different cavity gap and initial sealed gas pressure. The radius of the diaphragm is 1 mm, and the thickness is 5 μm.

For a pressure sensor with a silicon diaphragm with a radius of 1 mm and a thickness of 5 μm, the diaphragm deflection and cavity capacitance are calculated with different cavity gap and different initial sealing pressure using the large-deflection model as shown in Figures 2 and 3. The vacuum-sealed pressure sensor (*P_0_* = 0 kPa) will have a maximum downward deflection of 36.7 μm for a measurement range from 0 kPa to 120 kPa, which is the same as that of other gas-sealed pressure sensors, so a minimum gap of 37.0 μm is assumed for the vacuum-sealed pressure sensor at a pressure range from 0 kPa to 120 kPa. For the gas-sealed pressure sensors, the maximum downward deflection is effected by the cavity gap, which is less than the cavity gap. If the pressure of ambience is less than the pressure in the cavity, the diaphragm will deflect upward.

**Figure 4.**
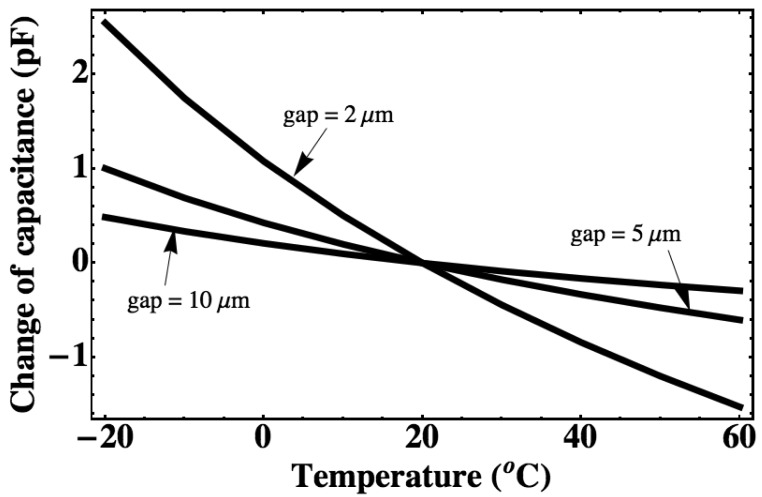
The relation of cavity capacitance change and temperature calculated by large-deflection model with different cavity gap and initial sealed gas pressure of 100 kPa. The radius of the diaphragm is 1 mm, and the thickness is 5 μm.

The capacitances of the all sensors mentioned in Figure 2 are calculated with the same measurement range (Figure 3). The pressure sensitivity can be expressed as the ratio of capacitance change to pressure change. It can be seen that the sensor sealed with 100 kPa gas can realize a higher sensitivity than the sensor sealed with vacuum since a very small gap is kept at around a 100 kPa. Inserting the Equations (1), (3), (6) and (7) in Equation (5), the deflection with different temperature can be obtained. For the vacuum-sealed pressure sensor, it will have no deflection change if the temperature changes because the pressure in the cavity is always 0 kPa. The thermal drift due to the thermal expansion of the gas inside of the cavity is calculated with different cavity gap using the above equations as shown in Figure 4. It is shown that for a gas-sealed pressure sensor, the narrower the gap, the higher the sensitivity and the larger the thermal drift will be.

### 2.2. Mechanical Temperature Compensation Structure

**Figure 5.**
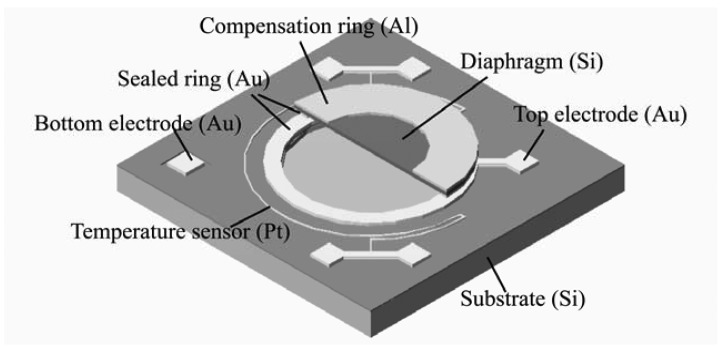
Schematic structure of the capacitive pressure sensor with temperature compensation ring.

**Figure 6.**
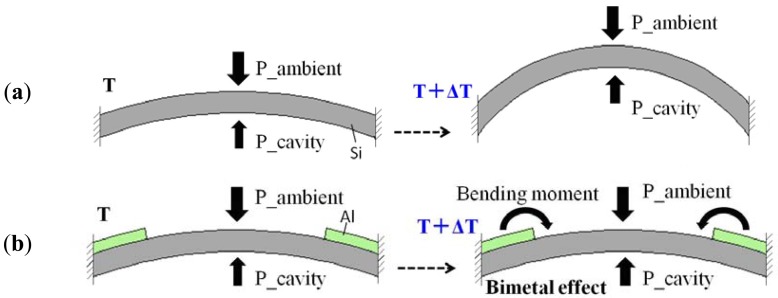
Temperature compensation mechanism. (**a**) diaphragm without compensation ring. (**b**) diaphragm with compensation ring.

In order to solve the thermal drift problem of the gas-sealed pressure sensor, a ring-shaped compensation structure with a thermal expansion coefficient higher than silicon is developed on the diaphragm surface (Figure 5). The compensation mechanism is shown in Figure 6. If the ambient pressure keeps constant, the diaphragm without compensation structure will bend upward due to gas expansion when the temperature rises from T to T + ΔT. However, the diaphragm with a higher thermal expansion coefficient material compensation ring (for example Al) will bend downward due to the bimetal effect when the temperature rises. If a compensation ring with thermal expansion coefficient larger than the silicon is on the surface of the diaphragm, or a compensation ring with thermal expansion coefficient less than the silicon on the backside of the diaphragm, the diaphragm will bend downward. On the other hand, if compensation ring with thermal expansion coefficient less than silicon is on the surface of the diaphragm, or a compensation ring with thermal expansion coefficient larger than the silicon is on the backside of diaphragm, the diaphragm will not bend downward. The deflection is related to the thickness of the compensation ring and the difference of thermal expansion coefficient between the silicon and the material of the compensation ring. The thicker the compensation ring, the larger the downward deflections will be; and the greater the difference, the larger the downward deflection. If the increase in capacitance caused by the bimetal effect is equal to the decrease in capacitance caused by the gas expansion when temperature rises, the thermal drift will be completely suppressed. The best compensation effect can be achieved by optimizing the dimensions of the compensation ring.

For the device with the compensation ring, If the temperature changes from *T_0_* to *T*, and the ambient pressure is constant, the change of volume and capacitance of the sealed cavity (*ΔV′* and *ΔC′*) are calculated by the Equations of (8) and (9), respectively:

(8) $$\Delta V\prime =V\prime -V_{0}^{\prime }=-\int_{0}^{r_{0}}2\pi r(\omega \prime (r)-\omega_{0}^{\prime }(r))dr$$

(9) $$\Delta C\prime =C\prime -C_{0}^{\prime }=\int_{0}^{r_{0}}\frac{{ɛ}_{0}{ɛ}_{r}2\pi r(\omega \prime (r)-\omega_{0}^{\prime }(r))}{\left(g-\omega_{0}^{\prime }(r))(g-\omega \prime (r)\right)}dr$$

where *ω′_0_*(*r*) is the deflection of diaphragm, *V′_0_* is the volume of sealed cavity, and *C′_0_* is the capacitance of the sealed cavity when the device is at the reference temperature of *T_0_* and the reference pressure of *P_0_*. When the temperature changes to *T*, the corresponding deflection, cavity volume and the capacitance change to *ω′*(*r*), *V′* and *C′*, respectively.

If the gap distance of the cavity is much larger than the deformation, the change of capacitance can be approximated by Equation (10), which indicates that the temperature compensation effect can be approximately expressed by the change of the sealed cavity volume:

(10) $$\Delta C^{\prime }\approx -\frac{{ɛ}_{0}{ɛ}_{r}}{g^{2}}\Delta V^{\prime }$$

When the temperature rises, the deflection of a diaphragm without compensation ring will be upward, so *ΔV′* > 0, *ΔC′* < *0*, and the capacitance will decrease. If a compensation ring with a thermal expansion coefficient larger than silicon is used on the surface of the diaphragm, the diaphragm will deflect downward, so *ΔV′* < 0, *ΔC′* > *0*, and the capacitance will increase, which can cancel out the capacitance change caused by gas expansion. Oppositely, if the diaphragm with a compensation structure deflects upward, so, *ΔV′* > *0*, *ΔC′* < *0*, the compensation structure has no temperature compensation effect.

The diaphragm deflection caused by the compensation ring is simulated with ANSYS using Shell181 elements. Aluminum is used as compensation structure material in the simulation. The material parameters of Young's modulus (*E*), thermal expansion coefficient (*α*), and Poisson's Ratio (*ν*) used for simulation are shown in Table 1.

**Figure 7.**
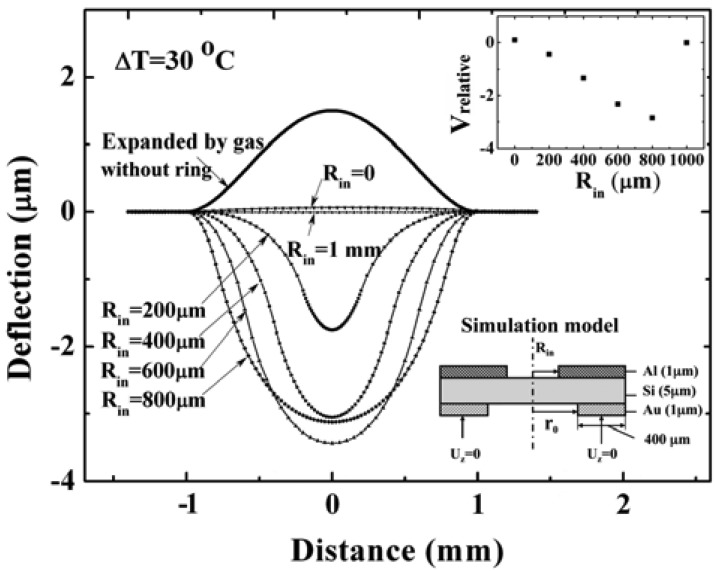
The deflection along diaphragm diameter without Al ring expanded by sealed gas, and the deflection with Al ring simulated with ANSYS. The below right is simulation model; the upper right is the normalizing volume change (with Al ring) relative to the volume change due to the expanded deflection. The temperature change is 30 °C, the radius (R) is 1 mm.

For a pressure sensor with a silicon diaphragm with a radius of 1 mm, a thickness of 5 μm, a gap of 5 μm, the model for simulation and the results are shown in Figure 7. The *V_relative_* in Figure 7 is the normalized volume change relative to the volume change due to the expanded deflection. When the temperature increases, the diaphragm without a compensation ring will bend upward due to the expansion of the sealed gas. In this case, the volume changes can be calculated by integrating the deflection profiles. The larger the *V_relative_* (absolute value) is, the greater the temperature compensation effect will be. As a result, it is found that even though the diaphragm with a ring of inner radius of 600 μm has the largest deflection at the center of the diaphragm, the diaphragm with an 800 μm-radius ring has the largest volume change. Namely, the ring with an inner radius of 800 μm generates a larger compensation effect. If the ring's inner radius decreases to zero (the diaphragm is fully covered by Al), the diaphragm will deflect upward. It is same as the deflection expanded by gas in the cavity. So, it has no compensation effect. Instead, it will increase the thermal drift. It is consistent with the bimetallic circular plate (the material with larger thermal expansion coefficient is on top) deflection when it is heated. The simulation result about compensation ring with inner radius of zero can also be verified by the theoretical calculation formula from Roark's formula for stress and strain [16].

**Figure 8.**
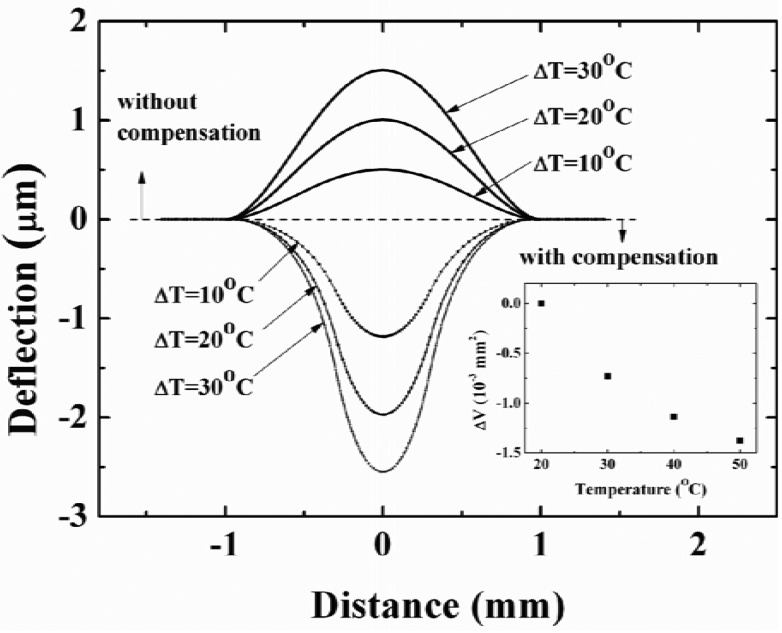
The deflection along diaphragm diameter without Al ring expanded by sealed gas, and the deflection with Al ring simulated with ANSYS. The below right is the volume change. The radius (R) is 1 mm, and the inner radius of compensation ring is 300 μm. Reference temperature is 20 °C.

The compensation effect with different temperature is also simulated for a diaphragm with compensation ring with inner radius of 300 μm. The simulation results are shown in Figure 8. The simulation model, device and material parameters are same as Figure 7. The reference temperature is 20 °C. The deflections expanded by gas without compensation structure are illustrated by upward convex curves, the deflections caused by compensation structure (cavity gas pressure change is not included) are illustrated by downward convex curves. We can integrate the deflection profiles to obtain the volume difference (Δ*V*) with/without compensation structure. Through Equation (10), the compensation effect can be compared to a certain extent. It is seen that the compensation effect becomes better with increasing temperature.

## 3. Fabrication

**Figure 9.**
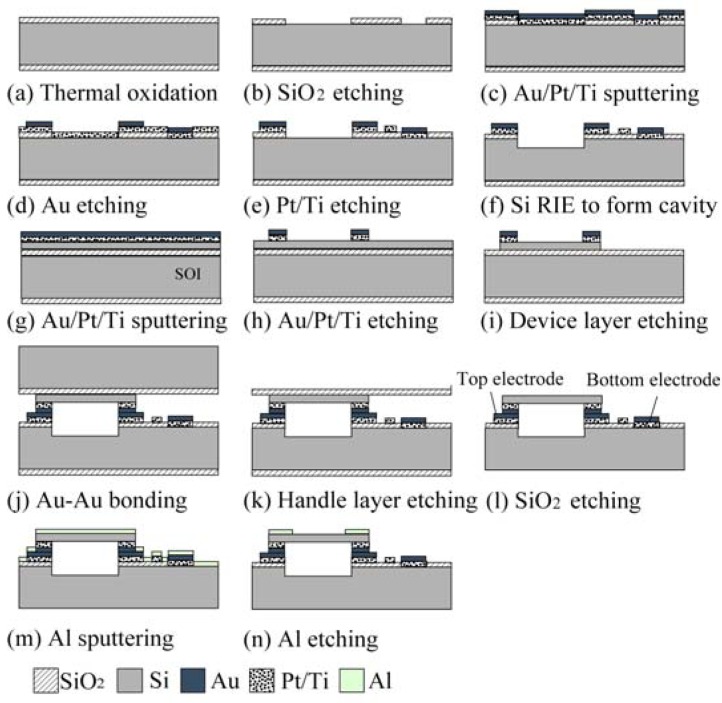
Fabrication process chart.

The compensation effect of the ring structure is verified by the following experiments. First, a pressure sensor without the Al compensation ring was fabricated with a diaphragm of radius 1 mm, an 8 μm gap for sensing capacitance, and an Au ring for bonding of 400 μm width. The main steps of the fabrication process are shown in Figure 9.

**Figure 10.**
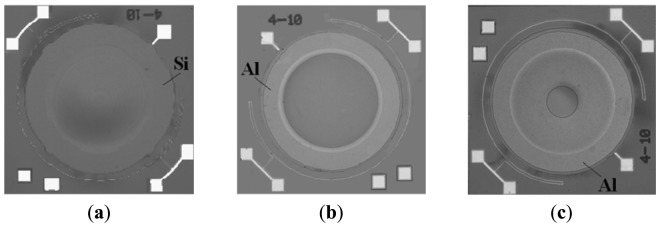
Micrograph photos of the fabricated devices: (**a**) no compensation ring; (**b**) compensation ring with an inside radius of 900 μm; (**c**) compensation ring with an inside radius of 300 μm.

For the substrate side, the starting material is a 4-inch silicon wafer with a resistivity less than 0.01 Ωcm. A SiO_2_ layer with a thickness of 1 μm is formed using thermal oxidation. A metal layer of Au (500 nm)/Pt (100 nm)/Ti (30 nm) is deposited by plasma sputtering, and patterned by reactive ion etching (RIE) to form the bonding layer. For the diaphragm side, the fabrication processes starts from a silicon-on-insulator (SOI) wafer. The thickness of the device layer, the buried oxide (BOX) layer, and the handle layer are 5 μm, 0.6 μm, and 400 μm, respectively. Metal layers of Au (500 nm)/Pt (50 nm)/Ti (30 nm) are deposited on the device layer of the SOI wafer and patterned by RIE to form the bonding layer and the top electrode. The substrate side and the diaphragm side are bonded to each other at 1 Atm of ambient nitrogen using the Au-Au diffusion bonding process at bonding temperature of 400 °C and bonding pressure of 5.5 MPa. Finally, the handle layer and the BOX layer of SOI are removed by deep RIE and BHF etching to complete the final device. The fabricated device is shown in Figure 10(a). After the capacitance-temperature characteristic, and capacitance-pressure characteristic are measured, an Al ring with thickness of 0.8 μm is additionally patterned on the diaphragm surface for compensation of the characteristics (Figure 9(m,n)). The fabricated devices with compensation ring with inner radius of 900 μm and 300 μm are shown in Figure 10(b,c).

## 4. Measurements

**Figure 11.**
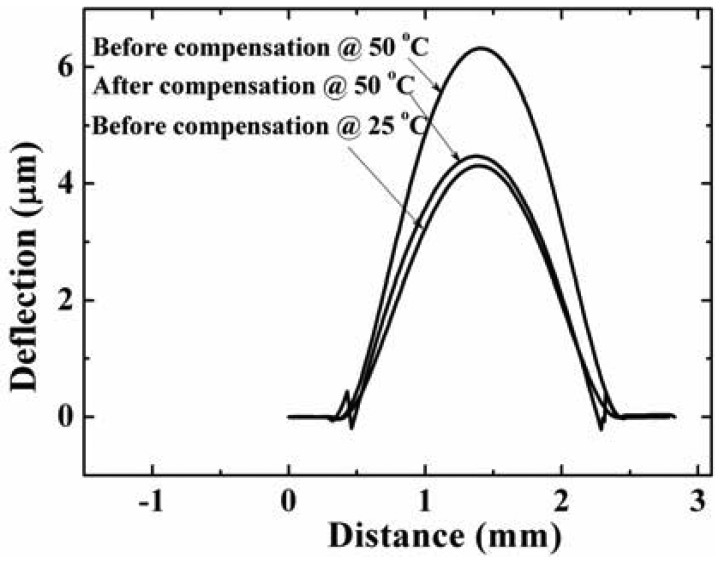
The deflection along diaphragm diameter without Al ring at 25 °C and 50 °C, and the deflection of diaphragm with Al ring with an inside radius of 900 μm at 50 °C. They are measured by white-light interferometer (Zygo^®^ NewView 6000, USA). The radius of the diaphragm is 1 mm (not include the sealed ring width), the gap is 8 μm, and the width of sealed ring is 400 μm.

The temperature characteristics of the fabricated devices shown in Figure 10 were measured. The deflection profiles of a device before and after compensation with compensation ring with an inner radius of 900 μm are measured by a white light interferometer (Zygo^®^ NewView 6000, USA) at 25 °C and 50 °C (Figure 11).

It is found that the diaphragm of the pressure sensor without compensation ring is convex at 25 °C due to the pressure difference between the sealed cavity and the ambient, and its deflection of diaphragm center at 25 °C and 50 °C are 4.2 μm and 6.3 μm, respectively. If the deflection *ω_max_* in Equation (3) is substituted by the value of 4.2 μm, according to the Equations of (3), (6), (7), and (8), the deflection of diaphragm center at 50 °C can be calculated. The calculated result is 6.0 μm. It is very closed to the measured result of 6.3 μm. The calculation result can be considered accurate if the temperature error and sensor sizes error are taken into account. It is also clearly shown that the deflection profile of the compensated sensor at 50 °C is very close to the initial deflection at 25 °C.

**Figure 12.**
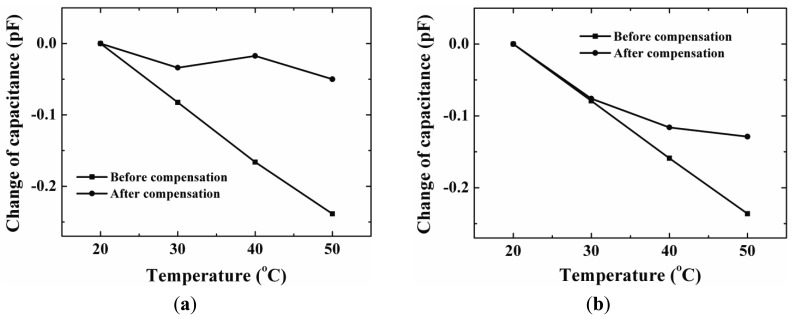
The capacitance-temperature characteristics before and after compensation with an Al ring: (**a**) the ring's inside radius is 900 μm; (**b**) the ring's inside radius is 300 μm.

The capacitance-temperature characteristics for both devices are also measured. A temperature of 20 °C is used as the reference temperature, and the capacitance at 20 °C is used as the reference capacitance. The difference between the measured capacitance and the reference capacitance against the temperature is plotted. The ratio of the capacitance change to the corresponding temperature change can be used to express the capacitance-temperature coefficient. The measured results of one device with a compensation ring with inner radius 900 μm are shown in Figure 12(a). It indicates that the capacitance-temperature coefficient is much reduced from 7.7 fF/°C to approximately zero after compensation. The other device's measurement results are shown in Figure 12(b). For this device, the inner radius of the compensation ring is 300 μm. It is shown that the compensation effect is not obvious when the temperature change is less than 10 °C. The larger the temperature change is, the more obvious the temperature compensation effect. This measured result can be proved by simulation result shown in Figure 8. Comparing Figure 12(a,b), it is found that the compensation effect of a compensation ring with inner radius of 900 μm is better than the compensation ring with inner radius of 300 μm. This also can verify the simulation results shown in Figure 7.

**Figure 13.**
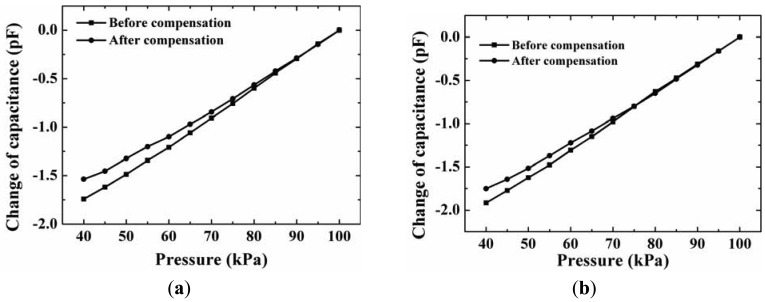
The capacitance-pressure characteristics before and after compensation with an Al ring: (**a**) the ring's inside radius is 900 μm; (**b**) the ring's inside radius is 300 μm.

In addition, the capacitance-pressure characteristics before and after compensation are measured at a temperature of 20 °C (Figure 13). The capacitance at atmospheric pressure of 100 kPa is used as the reference capacitance. The pressure sensitivity can be calculated by dividing the pressure change into the corresponding capacitance change. The measured pressure sensitivity of one device before and after compensation with a 900 μm inner radius ring is 29.2 fF/kPa and 25.0 fF/kPa in a pressure range from 40 kPa to 100 kPa. The pressure sensitivity of the other device before and after compensation with a 300 μm inner radius ring is 31.7 fF/kPa and 29.2 fF/kPa in a pressure range from 40 kPa to 100 kPa, respectively. For the two devices, in a pressure range from 80 kPa to 100 kPa, the sensitivities before and after compensation are almost same.

## 5. Conclusions

A gas-sealed capacitive pressure sensor with a mechanical temperature compensation structure has been successfully developed. The pressure sensor was fabricated by Au-Au diffusion bonding. The calculation model and the simulation results of the compensation ring structure and the compensation effect are verified by the fabrication and measurement results. The compensation ring structure with 900 μm inner radius has a better temperature compensation effect than the 300 μm inner radius ring, and it can realize a very lower temperature coefficient while maintaining almost the same sensitivity. It is possible to obtain a near-zero temperature coefficient gas-sealed capacitive pressure sensor by optimizing the sizes and material parameters of the temperature compensation ring. Future work will focus on the residual stress in the diaphragm.

## Figures and Tables

**Table 1.** Material parameters for simulation.

**Materials**	**E (GPa)**	**α (ppm/K)**	**ν**
**Si**	170	2.6	0.28
**Al**	70	23.1	0.35
**Au**	70	14.2	0.44
